# Beta-cell-specific C3 deficiency exacerbates metabolic dysregulation and insulin resistance in obesity

**DOI:** 10.1016/j.molmet.2025.102302

**Published:** 2025-12-11

**Authors:** Ben C. King, Lucie Colineau, Julia Slaby, Olga Kolodziej, Vaishnavi Dandavate, Robin Olsson, Malin Fex, Anna M. Blom

**Affiliations:** 1 Division for Protein Chemistry, Department of Translational Medicine, Lund University, Sweden; 2 Molecular Metabolism Unit, Department of Clinical Sciences Malmö, Lund University, Sweden

**Keywords:** C3, Beta-cell, Obesity, Insulin resistance

## Abstract

**Background:**

C3 is highly expressed in human and rodent pancreatic islets, which secrete insulin to regulate blood glucose homeostasis. We have previously shown that cytosolic C3 protects pancreatic beta-cells from stress, by allowing cytoprotective autophagy, and that the same intracellular pool of C3 also protects beta-cells from cytokine-induced apoptosis.

**Methods:**

We now generated a beta-cell specific C3 knockout mouse (beta-C3-KO) to test whether cell-intrinsic C3 is required for beta-cell function in a whole animal model. These mice were placed on high-fat diet (HFD), blood glucose and insulin measurements taken over time, and tissues examined at endpoint by qPCR and immunofluorescence.

**Results:**

While no differences were found between in baseline metabolic performance when comparing floxed controls and beta-C3KO mice, significant differences were found when mice were put on high-fat diet (HFD). Beta-C3-KO mice gained more weight, exhibited higher fasting blood glucose and insulin levels, and showed signs of adipose tissue inflammation and insulin resistance. Consistent with previous results showing that C3 alleviates beta-cell stress, increased amounts of unprocessed pro-insulin were found in the circulation of HFD-fed beta-C3-KO mice, as well as in islets from these mice. Beta-C3-KO HFD mouse islets also had a higher proportion of insulin staining, and isolated islets released more insulin *in vitro*.

**Conclusion:**

The interaction of increased insulin secretion and HFD leads to enhanced weight gain. Cell-intrinsic expression of C3 is important for optimal function of mouse pancreatic beta-cells under metabolic pressure *in vivo*.

## Highlights

• We investigated functions of protein C3 by creating a beta-cell specific C3 knockout mouse (Beta-C3-KO).
• We previously showed that C3–KO clonal beta-cells have decreased autophagy and increased insulin content and secretion.
• Beta-C3-KO mice gained more weight on high fat diet (HFD), with higher serum insulin and proinsulin levels.
• An interaction between increased insulin and high calory availability (HFD) could be responsible for weight gain.
• Obese patients may require monitoring if treated with C3 inhibitors, as beta-cell C3 disruption may worsen glucose control.

## 1 Introduction

Type 2 diabetes (T2D) originates in a lifestyle of overnutrition and inactivity combined with polygenetic risks. Obesity is associated with a low-level systemic chronic inflammation [1,2], and inflammation in turn can influence insulin resistance [3]. Beta-cells can compensate for this by increasing their mass and insulin production. However, prolonged exposure to such an environment leads to beta-cell dysfunction characterized by endoplasmic reticulum (ER) stress, mitochondrial and secretory dysfunctions [4,5]. Understanding the ability of beta-cells to respond to stress and increasing their survival and identity is a proposed strategy to delay or prevent T2D onset.

Obesity and T2D are characterized by low-grade inflammation measurable in the circulation and diverse tissues [1]. The complement system is a part of humoral innate immunity, composed of a network of serum proteins and a central component is the protein C3, found at high concentrations in serum. Increased serum concentration of C3 is a risk factor for T2D development [6] and is associated with insulin resistance, BMI, body fat percentage, waist circumference, and triglyceride concentrations [[7], [8], [9]]. This suggests a potential role for C3 in obesity, T2D, and associated cardiovascular diseases. Complement becomes activated in a proteolytic cascade, with complement activation products becoming deposited and triggering inflammation. Increased levels of complement proteins are enriched in isolated pancreatic islets at early stages of T2D development [10], further linking complement activation to the initiation of T2D.

As well as its role in inflammation, complement has other, non-canonical functions. In particular, there has been a recent focus on cell-autonomous signaling by complement activation products in the regulation of cellular metabolism, in which complement acts in local or autocrine fashions, or even entirely intracellularly; individual cells can express the required components for activation of complement within intracellular compartments [11,12]. Relevant to the diabetes research field, activation of the alternative pathway of complement leads to C3 cleavage and release of the bioactive peptide C3a, which augments glucose-stimulated insulin secretion from beta-cells via stimulation of the C3a receptor (C3aR) [[13], [14], [15]]. In adipocytes, C3aR-mediated signaling has also been shown to regulate energy consumption and thermogenesis [16], in a sex-dependent manner. These results demonstrate that complement is not only activated in the context of infection or inflammation, but that “tonic” complement activation regulates homeostatic functions of various tissues [17,18]. These two roles of complement do however represent a double-edged sword [19], whereby complement contributes to both homeostatic functions, but also to inflammation that could be detrimental to function in a chronic setting. Consequently, it is crucial to understand tissue- and cell-type specific actions of C3 and complement.

We have previously shown that C3 expression is increased in pancreatic islets from diabetic patients, as well as in islets from various T2D rodent models [20]. Human islet C3 expression also correlated positively with BMI and HbA1c. Moreover, we showed that C3 participates in cytoprotective autophagy in the INS-1 rat insulinoma cell line, through its interaction with ATG16L1 [20], and defective autophagic processes in C3–KO INS-1 cells also had increased insulin content and subsequent secretion. Islets from C3 knockout mice also showed accumulation of autophagy substrates [20]. C3 knockout clonal INS-1 beta-cells also underwent increased apoptosis when challenged with IL-1β *in vitro* [21]. In addition to this, beta-cell specific C3–KO mice also developed diabetes more rapidly when challenged *in vivo* with streptozotocin, consistent with the more rapid death of beta-cells lacking C3 [21]. We demonstrated an intracellular interaction of C3 with the beta-cell-expressed Fyn-related kinase (FRK) [22,23], thus showing that C3 has multiple modes of cytoprotection. ATG16L1 and FRK are both found within the cytosol, where we have also identified the presence of C3. To determine the form of C3 responsible for the cytoprotective effect, we generated gene-edited clonal beta-cells targeting the signal peptide of C3, resulting in clones that do not secrete C3, but retain it within the cytosol. Importantly, these clones had a rescued phenotype as compared to KO clones [20,21], indicating that cell-intrinsic and not secreted C3 was responsible for the cytoprotective effect.

Taken together, this suggests that C3 plays different roles depending on its cellular location: C3 within beta-cells appears to play a homeostatic cytoprotective role, while circulating C3 could promote inflammation and contribute to diabetes progression. This is important to understand in the context of the increasing success of the development of drugs targeting serum C3 for the treatment of inflammatory disease [24]. To test this cell-intrinsic role of C3 in pancreatic beta-cells *in vivo*, we created a beta cell-specific C3KO mouse using a flox/Cre model, and subjected this to a high-fat diet, commonly used as an animal model for human diet-induced obesity [25].

## 2 Research design and methods

### 2.1 Mice

C3-tdTomato reporter mice (Floxed C3^IRES-tdTomato^ C57BL/6N) were generously provided by Dr. Claudia Kemper (National Institute of Health, USA). RIP-Cre mice (Tg(Ins2-cre)23Herr) were provided by Malin Fex (Lund University, Sweden). Mice were bred heterozygously and resultant genotyped littermates were used for experiments. The inbred C57BL/6N status of the colony was confirmed by miniMUGA substrain-discriminating genome array [26] (TransNetyx, USA). All animal experiments were approved by the Malmö-Lund ethical committee under permit number 20069/2020. Mice were kept on a chow diet until starting defined diets at 8 weeks of age, when mice were put on High Fat Diet (#D12492) or matching Low Fat Diet (#D12450J) from Research Diets (USA). Weights were recorded and displayed in results. Blood samples and glucose measurements were taken during the day between 11:00 and 15:00. No adverse events to procedures were recorded. Mice were not subject to previous procedures other than those described in the results. Mice were kept under specific pathogen-free conditions as ensured by testing of sentinel animals housed in the same room. Mice were housed 4 per cage in closed cages with liberal access to food and water, at 22 °C with 12-hour light/dark cycles under humidity-controlled conditions. Mice were randomly assigned to cages, and cages contained mixed genotypes. Samples were taken from mice one cage at a time, in random order. The main 6-month diet experiment was repeated twice, firstly with 8 animals per group, and a second time with a smaller number of animals for verification.

### 2.2 Glucose homeostasis measurements

Blood glucose levels were measured from the saphenous vein using the Accu-Chek Aviva Glucose meter and test strips (Roche, Switzerland). Before glucose or insulin challenge, mice were fasted by removal of food for 4 h. For the Intraperitoneal Glucose Tolerance Test (IPGTT), mice were injected intraperitoneally (IP) with glucose (1 g/kg of body weight). Blood samples were taken from the saphenous vein at given time points and glucose levels measured using the Accu-Chek Aviva Glucose meter and test strips (Roche). Blood samples were collected and serum prepared and stored for insulin measurements. For IP Insulin tolerance test (IPITT), mice were injected intraperitoneally with insulin (0.5 U/kg of body weight). Blood samples were taken from the saphenous vein and glucose levels were measured. All work was carried out in the animal facility for the department of Translational Medicine, Lund University, in Malmö.

### 2.3 Serum protein ELISAs

Serum was collected by resting freshly drawn blood at room temperature for 20 min, then on ice for 15 min, before spinning at 7500 xG for 10 min at 4 °C. Serum aliquots were stored at −80 °C. Insulin, pro-insulin, complement factor D/Adipsin, and C3 were measured using ELISA kits 10-1249-01 and 10-1232-01 (Mercodia, Sweden), ELISA kit DY5430 (R&D systems, USA), and ELISA kit ab263884 (Abcam, UK), respectively.

### 2.4 Homeostasis model assessment of insulin resistance (HOMA)

The homeostasis model assessment was used to estimate beta cell function and insulin sensitivity using fasting insulin and glucose measurements. The HOMA2 Calculator from the University of Oxford was used for calculations (http://www.dtu.ox.ac.uk/homacalculator). Quantitative Insulin Sensitivity Check Index (QUICKI) [27] was also used to measure insulin sensitivity as follows: QUICKI = 1/[log(I_0_) + log(G_0_)], where I_0_ is the fasting insulin, and G_0_ is the fasting glucose.

### 2.5 Gene expression by RT-qPCR

RNA was isolated from adipose tissue using RNeasy lipid tissue mini kit (QIAGEN, Netherlands). RNA concentrations were measured by Nanodrop and quality assessed by ScreenTape assay (Agilent, USA). Reverse Transcription was performed using Superscript IV Reverse Transcriptase (ThermoFisher Scientific, USA) following the manufacturer’s instructions. RT-qPCR was performed on cDNA using TaqMan Gene Expression Master Mix (ThermoFisher Scientific, #4369016), and Gene Expression Assays following the manufacturer’s instructions. The following Gene Expression Assays were used: Complement factor D (*Cfd*) Mm01143935_g1; Complement factor B (*Cfb*) Mm00433909_m1; *C3* Mn01232779_m1; *Cd163* Mm00474091_m1; *Adgre1* (F4/80) Mm00802529_m1; *Ccl2* (Mcp-1) Mm00441242_m1; *Itgam* (CD11b) Mm00434455_m1; *Il1β* Mm00434228_m1; *Tnfa* Mm00443258_m1; *Il6* Mm00446190_m1; Reference genes were *Hprt* Mm01545399_m1 and *B2m* Mm00437762_m1. RT-qPCR was run on the Viia 7 Real-Time PCR system and analyzed using the ΔCt method against both reference genes.

### 2.6 Immunofluorescence imaging

Fragments of pancreas and adipose tissues (visceral parametrial and subcutaneous inguinal depots) were fixed in 4% PFA in PBS overnight at 4 °C. Tissues were dehydrated and mounted in paraffin blocks. Sections of 5 μm were dewaxed and rehydrated, and a decloaking chamber used for antigen retrieval (90 °C for 10 min), in antigen retrieval buffer (citrate buffer: 10 mM sodium citrate, 0.05% Tween 20, pH 6.0). Tissues were analyzed from all available samples from each experimental group, where technically possible, as indicated in figures. For adipose tissue: sections were permeabilized with 0.2% Triton X-100 in PBS for 10 min and blocked in 2% BSA in PBS for 1h. Primary antibodies rabbit anti-perilipin (Abcam ab3526, 1:500) and rat anti-F4/80 (Abcam ab6640, 1:100) were diluted in 0.1% BSA in PBS, and incubated overnight at 4 °C. Slides were washed with PBS three times. Secondary antibodies Alexa Fluor 647 F(ab')_2_ Fragment Donkey Anti-Rabbit IgG (Jackson ImmunoResearch, USA, #711-606-152) and Alexa Fluor 488 F(ab')_2_ Fragment Donkey Anti-Rat IgG (Jackson ImmunoResearch #712-546-153) were diluted 1:500 in 0.1% BSA in PBS. Slides were incubated for 4hr at 4 °C. The slides were washed with PBS three times and mounted with DAPI-containing mounting media. For pancreatic sections: sections were blocked and permeabilized with 5% normal donkey serum in 5% Triton X-100 in PBS for 2 h at 4 °C. Guinea pig anti-insulin (Progen Biotechnik GmbH, Germany, #16049; 1:200) and rabbit anti-glucagon (ThermoFisher Scientific #15954-1-AP; 1:500) were diluted in blocking buffer. Slides were incubated overnight at 4 °C. Sections were washed three times with of PBS +5% Triton X-100. Secondary antibodies Cy2 Donkey Anti-Guinea Pig IgG (H + L) (Jackson ImmunoResearch #706-225-148) and Alexa Fluor 647 F(ab')_2_ Fragment Donkey Anti-Rabbit IgG (Jackson ImmunoResearch, #711-606-152) were diluted 1:500 in blocking buffer for 4hr at 4 °C. Sections were washed three times with of PBS +5% Triton X-100. The slides were washed with PBS three times and mounted with DAPI-containing mounting media. For tdTomato staining, pancreatic sections were incubated 15 min in 0.25% (w/v) Sudan black B (Sigma Aldrich, Germany) in 70% propanol, washed in 70% propanol, washed four times in PBS and blocked in 5% BSA for 2 h at 4 °C. Goat anti-tdTomato (OriGene Technologies GmbH, Germany, #AB8181-200; 1:50) was diluted in blocking buffer. Slides were incubated overnight at 4 °C. Sections were washed three times with of PBS +5% Triton X-100. Secondary antibody Alexa Fluor 647 F(ab')_2_ Fragment Donkey Anti-Goat IgG (Jackson ImmunoResearch, #705-606-147) was diluted 1:500 in blocking buffer for 4hr at 4 °C. Sections were washed three times with of PBS +5% Triton X-100 then mounted as described above. For pro-insulin staining, AlexaFluor647-labeled anti-pro-insulin (R&D Systems, #IC13361R, 1:100) was incubated overnight at 4 °C, in combination with glucagon staining, before washing and mounting as described above. Slides were imaged with a Zeiss (Germany) LSM 510 Meta Confocal microscope. Images were analyzed using Ilastik software [28] for objective capture of numbers and sizes of cells, as well as islet sizes and areas of insulin/pro-insulin staining. All image analysis was performed blinded to sample identity. Multiple sections or fields were used per animal, as technical repeats.

### 2.7 Pancreatic islet isolation

Mice were euthanised and the main pancreatic duct clamped at the duodenum. The pancreas was perfused via the common bile duct with ice-cold 0.5 mg/ml collagenase (Sigma–Aldrich, #C9263) in HBSS, then removed and placed on ice. The perfused pancreas was then incubated at 37 °C for 17 min, washed with ice-cold HBSS and shaken vigorously, before placing on ice for 4 min to allow islets to settle. The supernatant was removed and washing was repeated 4 times. Then, islets were isolated by picking them under a stereo-microscope. Inherent C3 reporter tdTomato expression in fresh live islets was imaged using a BioTek Cytation 5 instrument (Agilent).

### 2.8 Islet insulin secretion assay

Isolated mouse islets were picked directly into pre-warmed RPMI with 10% FBS and incubated in a cell culture incubator for 30 min for recovery, before transferring to secretion assay buffer (SAB) (114 mM NaCl, 4.7 mM KCl, 1.2 mM KH_2_PO_4_, 1.16 mM MgSO_4_, 20 mM HEPES, 2.5 mM CaCl_2_, 25 mM NaHCO_3_, pH 7.2, 0.1% BSA) with low glucose (2.8 mM) for 1 h to stabilize, then 5 islets were transferred per well of a 96-well plate containing 200 μl of either low glucose (2.8 mM) or high glucose (16.7 mM) SAB. Six wells were used per condition per mouse. After 15 min in a 37 °C cell culture incubator, 75 μl supernatant was carefully removed to assess first-phase secretion. After 60 min, another 75 μl supernatant was removed. Islets and supernatants were stored frozen until measuring insulin content of lysates (after the addition of RIPA buffer and subsequent dilution into ELISA assay buffer) and supernatants, using Mercodia mouse insulin ELISA (10-1247-10).

### 2.9 Cell culture and C3 incubation

INS-1 832/13 cells were gene-edited to produce C3–KO clones as previously described [20]. Human-C3 over-expressing clones were produced by stably transfecting C3–KO INS-1 clones with pSELECT plasmid containing cloned human C3 cDNA, and verified by Western blotting and human C3 ELISA of supernatants. INS-1 clones were maintained in RPMI supplemented with 10% fetal calf serum, HEPES, 2 mM l-glutamine, 1 mM sodium pyruvate, and 50 mM beta-2 mercaptoethanol (Sigma). Clones were tested each month to ensure that they were free of mycoplasma infection. To assess C3 uptake, C3–KO INS-1 832/13 clones were incubated with or without added purified human C3 (Complement Technologies Inc., USA) or supernatant from C3-secreting clones, before washing and fractionation using the ProteoExtract Subcellular Proteome extraction kit (Sigma–Aldrich). Fraction samples were Western blotted using antibodies against C3 (Calbiochem, USA, #204869), with controls of beta-tubulin (Abcam, #ab6046, 1:10,000), protein disulfide isomerase (Enzo Life Sciences, USA, #ADI-SPA-891, 1:1000) and histone 2B (Abcam, #ab1790, 1:50,000) for cytosolic, membrane/organelle, and nuclear fractions respectively. Antibodies were validated using knock-out cell control samples (for C3) or by negative signals in fractions lacking those antigens.

### 2.10 Western blotting

Supernatants or subcellular fractions (prepared using the Mem-Per Plus eukaryotic protein extraction kit, ThermoFisher Scientific) were boiled in Laemmli buffer for 10 min at 95 °C and then run on 4–15% gradient SDS polyacrylamide gels, before transfer to PVDF membranes using BioRad turboblot transfer cells. Western blot experiments were performed 3 times.

### 2.11 Electron microscopy

Pancreatic islets were isolated from mice as described above, and then fixed for 3 h in 2% formaldehyde and 2% glutaraldehyde in 0.1M Sörensen phosphate buffer, before washing and preparing for electron microscopy at the Lund University Bioimaging Centre. Images were taken blinded using a Thermo Fisher Talos L120C transmission electron microscope.

### 2.12 Data and statistical analyses

Results displayed show mean and standard deviation unless otherwise indicated in figure legends. Where appropriate, superplots [29] are used to display total individual data points (e.g. individual islet values, small icons), as well as average values per mouse (large icons) to accurately display data spread. The experimental unit used for statistical analysis was a single animal, or the average value from several sections/islets from each animal, with exception of the mixed model analysis described below. Analysis of immunofluorescence was carried out blinded to the individual who analysed the stainings. Statistical analyses were carried out using GraphPad Prism version 10, using one-way or two-way ANOVA with Tukey post-test, unless otherwise indicated in figure legends. Statistics were carried out on average values per mouse, using multiple samples per mouse as technical replicates, unless otherwise stated. In Figure 6B,C, statistics were carried out using a linear mixed model analysis in R 4.3.3 with the Ime4 package. The following model specifications were used for the insulin staining: Imer (Insulinstaining ∼ Genotype + (1|MouseNumber), REML = FALSE, control = lmerControl (‘bobyqa’), data = MixedModel). A dummy variable was assigned, with 0 for the flox-control, and 1 for the beta-C3-KO genotype. A likelihood ratio test (LRT) was used to see if the full model was significantly better than the null model (Alfa = 0.05), and the 95 % confidence interval was extracted to analyse the variation. For the islet size the following model specifications were used: lmer (Isletsize ∼ Diet + (1|MouseNumber), REML = FALSE, control = lmerControl (‘bobyqa’), data = MixedModel). For this analysis, the dependent variable (islet size) was log transformed with the natural log to satisfy the model assumption of normally distributed residuals. After the analysis, the exponent for the value for the estimate of the fixed effect and confidence interval were calculated in R to get the multiplicative factor increase (in %) per 1 unit of the fixed effect. Both genotype and diet together and alone were tested for both analyses, but only the significant models are specified above. The model assumption of normally distributed residuals was assessed by plotting them against a normal distribution curve. The LRT assumes a Chi-square distribution, and that the restricted (residual) maximum likelihood (REML) is set to false. We assumed fixed slopes and random intercepts for the linear mixed model since that is the standard assumption, and we had no theoretical reason to believe otherwise. The control used was BOBYQA, which performs a derivative-free bound-constrained optimization using an iteratively constructed quadratic approximation for the objective function, which is used to optimize the model’s performance. For all graphs, ∗, *p* < 0.05, ∗∗, *p* < 0.01, ∗∗∗, *p* < 0.001, ∗∗∗∗, *p* < 0.0001.

## 3 Results

### 3.1 Generation and verification of beta-cell specific C3-knockout mice

**Figure 1:**
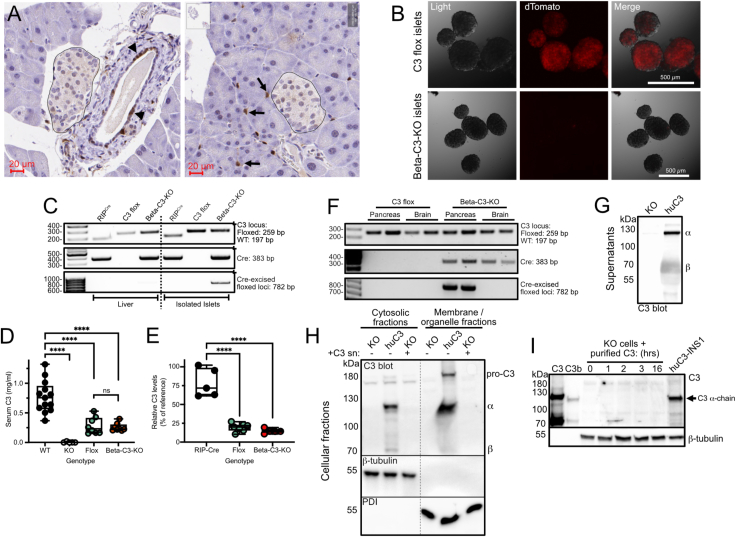
C3 is expressed in pancreatic islets of floxed C3 tdTomato reporter mice, and knocked out from β-cells by expression of RIP^Cre^. A) Pancreatic sections from C3 flox tdTomato reporter mice were labelled with anti-dTomato antibodies for immunohistochemistry. Islets are outlined for clarity. Arrow heads, ductal cells (left panel), small arrows, macrophages (right panel). B) Islets were freshly isolated from RIP^Cre^ and beta-C3-KO mice and imaged directly for tdTomato fluorescence. C) Genomic DNA was isolated from isolated islets or liver of RIP^Cre^, C3-flox and beta-C3-KO mice and PCR analysis run for C3 (top), Cre (middle) and Cre excised floxed loci (bottom). D) C3 ELISA measurements of serum collected from C3 flox or beta-C3-KO mice, or WT C57Bl/6N and total C3 KO controls. E) C3 ELISA measurements of serum from RIP^Cre^, C3-flox and beta C3–KO mice, compared to WT C57Bl/6N mouse serum as reference. F) Genotyping PCR results from pancreas and hypothalamus taken from C3-flox and beta C3–KO mice. G) Clonal C3–KO INS-1 β-cells (KO) or those stably expressing human C3 (huC3) were assessed for C3 secretion into supernatants by Western blot. H) Supernatant (sn) from huC3 cells were then incubated overnight with KO cells, before washing and fractionating cells for assessing intracellular C3 uptake, using huC3 cell fractions as positive control. I) Similarly, KO cells were incubated for increasing times with 100 μg/ml purified human C3, before washing cells and blotting whole lysates to assess uptake, with purified C3/C3b, and huC3 lysate as positive controls. No uptake or internalisation of C3 was seen. Blots, gel and images, representative from at least 3 independent repeats. D, E, show mean, SD, and range, each spot represents mean value for 1 mouse. Statistics in D, E, one-way ANOVA.

Our mouse model was created by crossing floxed C3 tdTomato reporter mice [30] with Tg(Ins2-cre)23Herr mice on the same background, expressing Cre recombinase under the control of the rat *Ins2* promoter [31]. The resultant beta-cell specific C3 KO mice are referred to in this paper as beta-C3-KO. We previously showed that C3 is expressed in pancreatic islets and is upregulated in islets from diabetic rodents and human donors [20]. Consistent with this, pancreatic islets from the tdTomato floxed C3-reporter mouse also stained positive for tdTomato (Figure 1A). C3 reporter signal was also strong in ductal epithelial cells and macrophages within the exocrine tissue. As expected, fluorescence was directly detectable in freshly isolated islets from floxed C3 reporter mice (Figure 1B). Beta-C3KO mouse islets completely lacked tdTomato fluorescence, demonstrating deletion of the C3 locus in beta-cells (Figure 1B), and confirming beta-cells as the main islet endocrine cells expressing C3. This was verified by PCR from isolated tissues, showing C3 deletion in isolated islets, but not in the liver, the main site of production of circulating plasma C3 (Figure 1C). Consequently, there was no difference in C3 levels in serum of control flox-C3 mice and beta-C3-KO mice on chow diet, at 10–12 weeks of age (Figure 1D), both having roughly 250 μg/ml of serum C3. A significant reduction was, however, seen between both the floxed and beta-C3-KO mice compared to non-floxed WT mice on the same C57Bl/6N background (Figure 1D), indicating that the introduction of the flox sites and the tdTomato reporter may partly inhibit translation of the C3 protein. Consistent with this, there was also a significant reduction in serum C3 in floxed and beta-C3-KO mice, compared to RIP-Cre mice (Figure 1E). Due to demonstrated systemic roles of C3 and complement activation in affecting metabolism at other sites [19], we therefore decided not to use RIP-Cre mice as comparable controls. Insulin promoter-driven Cre expression has been reported in the brain, and complement mediated synaptic pruning [32] could conceivably affect central regulation of appetite or metabolism, but we could find no evidence of cre-mediated deletion of the C3 locus in isolated beta-C3-KO mouse hypothalamus (Figure 1F), the area of the brain most involved in metabolic regulation, nor in wider areas of the brain (data not shown).

The beta-C3-KO mice therefore lacked pancreatic islet expression of C3 but still had abundant levels of C3 in circulation. To investigate whether beta-cells can take up C3 from the extracellular environment, as has been shown with some other cell types [33,34], C3–KO INS-1 clonal beta-cells were compared with clones stably expressing human C3, as demonstrated by Western blot of supernatants (Figure 1G). C3–KO cells incubated overnight with C3-containing supernatants did not internalize any C3, as detectable by Western blot of lysates (Figure 1H), nor when 100 μg/ml serum-purified C3 was added to supernatants (Figure 1I). Therefore, with no uptake of C3 by beta-cells, it can be deduced that any changes in the function of C3-knockout beta-cells within mice that still have abundant serum C3 levels, would likely be due to cell-intrinsic intracellular C3.

### 3.2 Beta-C3-KO mice have no phenotypic difference on chow diet

**Figure 2:**
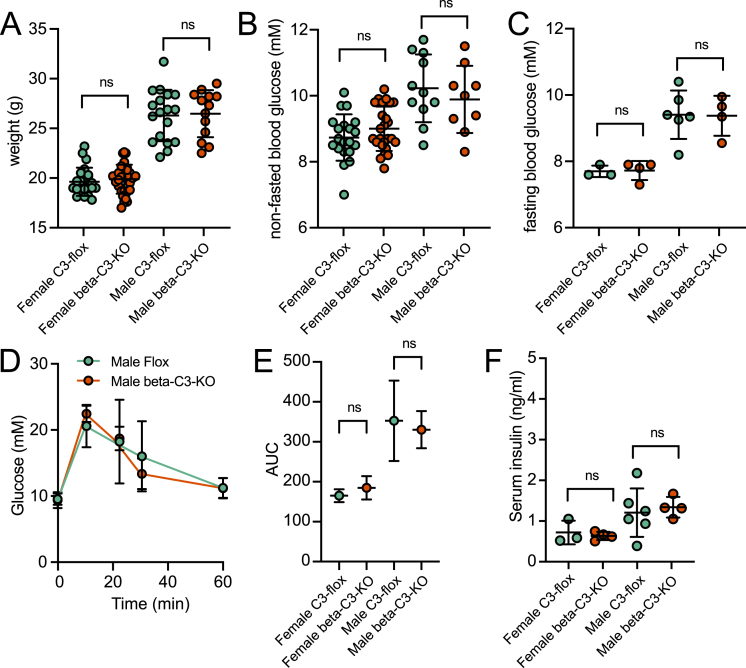
Beta-cell specific C3 deletion does not affect basal metabolism in healthy mice. C3 flox and beta-C3-KO mice on chow diet were compared for A) body weight, B) blood glucose levels and C) fasting blood glucose levels, at 8–9 weeks of age. D) At 10 weeks of age, male mice were subjected to IPGTT (*n* = 3–4 per group). E) AUC for IPGTT in both male and female mice. F) Fasting serum insulin levels in 8–9-week-old male and female mice. 2-way ANOVA statistical test was applied in each panel.

We next assessed baseline differences in islet function and blood glucose homeostasis between the C3-floxed control and beta-C3-KO mice. At 8–9 weeks of age, there was no genotype-dependent difference in males or females in body weight (Figure 2A), non-fasted or fasted blood glucose (Figure 2B,C), or fasting serum insulin levels (Figure 2D). Similarly, there was no difference in IPGTT responses (Figure 2E,F), showing that under basal conditions, lack of C3 expression in pancreatic beta-cells results in no functional difference in blood glucose homeostasis.

### 3.3 Beta-C3-KO mice have a diabetes-prone phenotype on a high-fat diet

**Figure 3:**
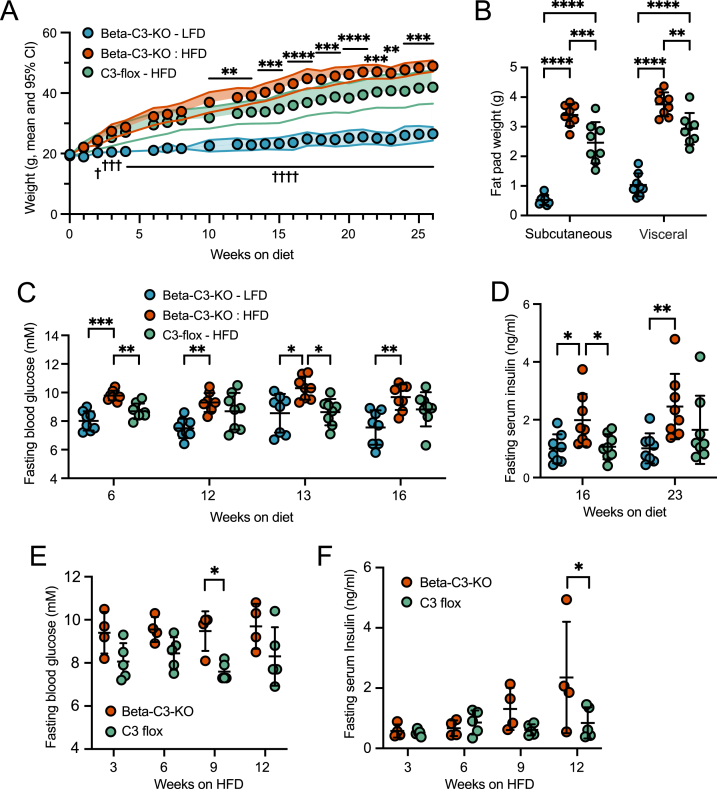
Female beta-C3-KO and control C3-Flox mice (8 per group) were placed on LFD and (HFD) at 8–9 weeks of age, and weight tracked over time (A). B) Subcutaneous (inguinal) and visceral (parametrial) fat pads were weighed at the endpoint. C) Fasting blood glucose levels were also measured at given time points. D) Serum was also collected from fasted mice and insulin levels measured by ELISA. E) In a second repeat of the experiment, a new group of female beta-C3-KO and C3-Flox mice were put on HFD at 8–9 weeks of age, and fasting blood glucose (E) and serum insulin (F) were measured. Statistics, 2-way ANOVA. In (A), lines indicate mean and 95% confidence intervals; stars indicate statistically significant differences between HFD C3 flox and beta-C3-KO mice, while crosses indicate statistically significant differences between LFD mice and HFD mice.

We previously showed that C3–KO INS-1 beta-cells did not have reduced basal survival when unchallenged, despite defects in autophagic function [20]. However, C3–KO INS-1 cells displayed increased apoptosis when placed under various stressors, such as glucolipotoxicity or inflammatory cytokines [20,21]. We therefore placed control and beta-C3-KO mice on HFD for 25 weeks. All mice on HFD increased in body weight compared to control mice on a low-fat control diet (LFD). HFD beta-C3-KO mice also showed significantly greater weight gain than HFD C3-flox control mice (Figure 3A). This was reflected in the significantly greater mass of both inguinal (subcutaneous) and parametrial (visceral) fat pads in the HFD beta-C3-KO mice (Figure 3B). At multiple time points, fasting blood glucose was also significantly higher in the HFD beta-C3-KO mice compared to HFD C3-flox mice (Figure 3C). In addition, beta-C3-KO mice also developed higher fasting serum insulin (Figure 3D), suggesting compensatory hypersecretion of insulin to overcome insulin resistance.

We repeated this experiment, focusing on the early time points of HFD to identify when homeostasis becomes dysregulated in the KO mice. Although this second cohort was underpowered (*n* = 4 to 5 per group), there was a tendency for increased blood glucose in beta-C3-KO mice compared to control mice at all time points tested, which reached significance at 9 weeks of diet (Figure 3E). Insulin levels were the same between the two groups at 3 and 6 weeks of HFD but became significantly increased in the beta-C3-KO mice at 12 weeks (Figure 3F).

### 3.4 Beta-C3-KO mice have increased adiposity and adipose inflammation

**Figure 4:**
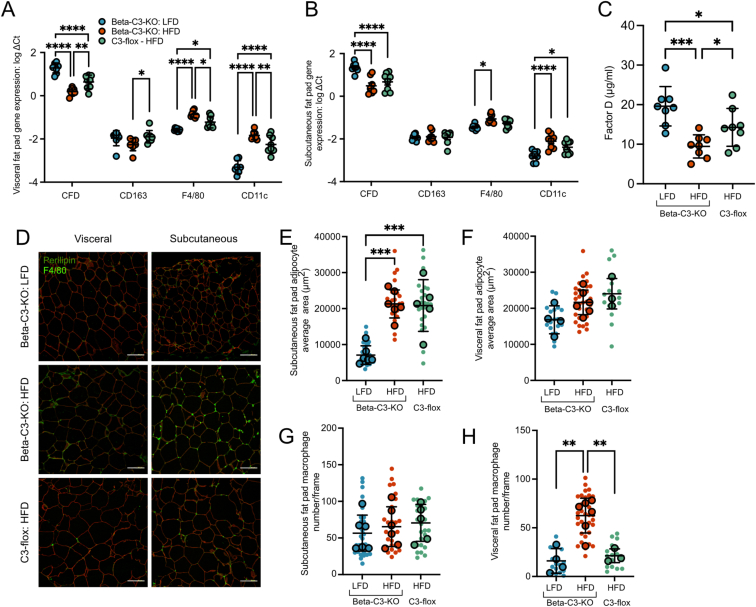
Gene expression in A) visceral (parametrial) fat pad and B) subcutaneous (inguinal) fat pads of mice. C) Serum CFD levels of the same mice, as measured by ELISA. D) Adipose tissues from mice were stained for perilipin (red) and macrophage marker F4/80 (green). E) Average adipocyte sizes were then calculated in subcutaneous (E) and visceral (F) fat pads, as well as macrophage numbers in subcutaneous (G) and visceral (H) fat pads. Statistics in A, B: 2-way ANOVA, with gene of interest and mouse genotype being two independent variables. Data was tested for difference in gene expression between mouse genotypes. In C, F, G: one-way ANOVA. Scale bars in D), 100 μm.

We next analyzed both visceral (parametrial) and subcutaneous (inguinal) adipose tissue depots. RT-qPCR assays showed an increase in inflammatory macrophage markers (CD11c and F4/80) in HFD compared to LFD mice, and this increase was significantly higher in beta-C3-KO HFD mice compared to control HFD mice, in visceral fat (Figure 4A). Interestingly, beta-C3-KO HFD mice showed no change in expression of CD163 in visceral fat, a marker of M2 macrophages, whereas this marker was upregulated in visceral fat of C3-flox HFD mice. These results indicate a skew towards a more inflammatory macrophage population in visceral fat of beta-C3-KO mice compared to C3-flox mice, on HFD. In subcutaneous fat, the upregulation of F4/80 and CD11c relative to LFD controls reached stronger significance in beta-C3-KO HFD mice than in C3-flox HFD mice, although there was no significant difference between the two HFD groups (Figure 4B). In addition, expression of complement factor D (FD), an adipokine also known as adipsin, was decreased in both HFD adipose tissues, compared to LFD controls, as previously described [35]. Downregulation of CFD was significantly greater in HFD beta-C3-KO mouse visceral fat than in HFD flox control mice (Figure 4A). This reduction in expression also translated into significantly lower serum levels of CFD in HFD beta-C3-KO mice than in HFD controls (Figure 4C). Obesity in the HFD mice was reflected by significantly larger adipocyte size in subcutaneous inguinal fat pads (Figure 4D,E). Visceral fat adipocyte size trended towards increased size under HFD but this did not reach significance compared to LFD mice (Figure 4F). Changes in numbers of macrophages observed by F4/80 immunofluorescence did not reach significance in subcutaneous far (Figure 4G), but a significant increase in macrophages was observed in visceral fat of Beta-C3-KO HFD mice in comparison to either LFD controls or HFD C3-flox mice (Figure 4H). This was in agreement with increased RT-qPCR measurement of F4/80 expression (Figure 4A), indicating increased inflammatory macrophage infiltration, a property that is associated with increased adipose tissue insulin resistance [36].

### 3.5 HFD beta-C3-KO and floxed mice are insulin resistant

**Figure 5:**
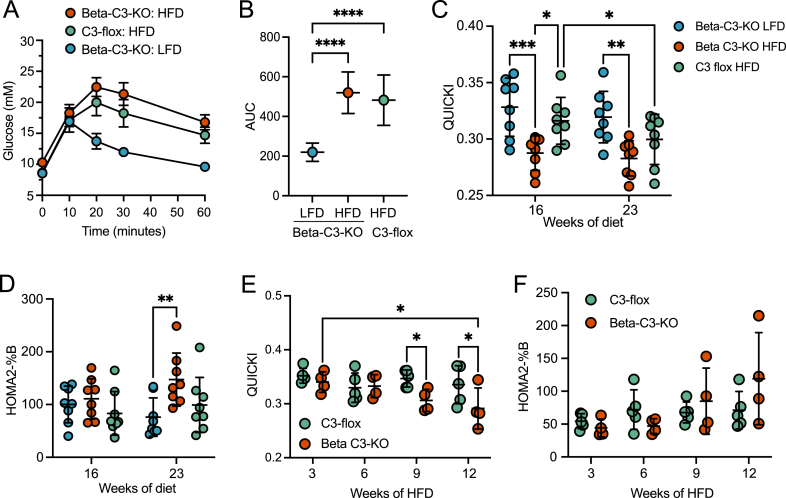
A) C3-Flox and beta-C3-KO mice on HFD for 13 weeks, and LFD controls, were challenged by IPGTT and blood glucose measurements taken over time. B) Area under the curves (AUC) were calculated for data shown in (A). QUICKI (C) and HOMA-%B (D) (measures of Insulin sensitivity and beta-cell activity respectively) were calculated for individual mice as described in the methods. In a second, smaller cohort of mice on HFD, QUICKI (E) and HOMA2-%B (F) were also calculated in HFD C3-flox and Beta-C3-KO mice only, at earlier time points, showing similar results. Statistics, 2-way ANOVA, except B, one-way ANOVA.

During the IPGTT, both HFD groups displayed delayed glucose clearance, as compared to LFD fed mice (Figure 5A,B). HFD beta-C3-KO mice were not significantly different from control HFD floxed mice, as observed by the area under the curves between the two HFD groups (Figure 5B). Calculations of insulin sensitivity (QUICKI) (Figure 5C) and beta-cell function (HOMA2-%B) (Figure 5D) [37] did, however, reveal lowered insulin sensitivity already at 16 weeks in beta-C3-KO compared to control mice on HFD, and subsequently increased compensatory beta-cell function in beta-C3-KO compared to control mice on HFD becoming significant at 23 weeks. In a second experimental cohort of mice, focusing on earlier time points, a significant decrease in the QUICKI measurement of insulin sensitivity developed with time in HFD fed beta-C3-KO mice, and became significantly worse compared to control HFD mice already at 9 weeks (Figure 5E), while HOMA2-%B showed a trend towards an increase in the HFD fed beta-C3-KO mice at 9 and 12 weeks (Figure 5F). This measurement of beta-cell activity did not, however, reach significance in this second cohort, possibly due to the earlier time points and smaller group sizes.

### 3.6 Beta-C3-KO HFD mice have increased insulin and pro-insulin content

**Figure 6:**
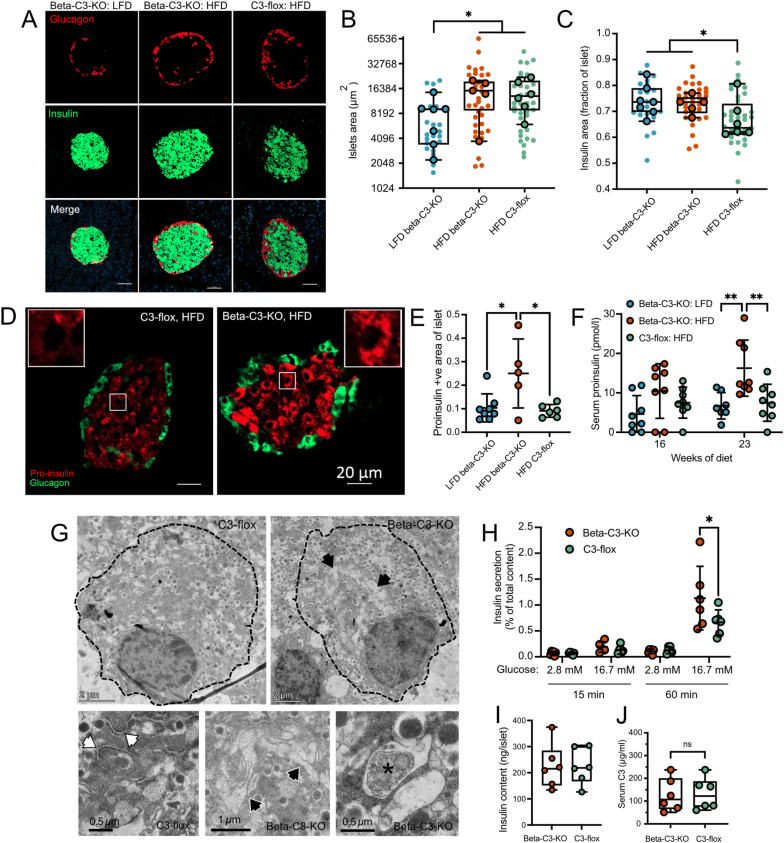
A) Pancreas sections from treated mice were stained for glucagon (green) and insulin (red) to visualize islets and assess their size. Blue: DAPI. Scale bars, 50 μm. B) Quantification of total islet size in sections from 22 islets from pancreas sections of 7 LFD beta-C3-KO mice, 33 islets from pancreas sections of 5 HFD beta-C3-KO mice, and 35 islets from pancreas sections of 6 HFD C3-flox mice. C) Fraction of total islet staining for insulin, from the same islets analysed in (B). D) Examples of staining of individual HFD islets for glucagon (green) and pro-insulin (red). E) Quantification of pro-insulin staining in islets from each group of mice. F) Quantification of pro-insulin in serum of mice at given time points, measured by pro-insulin specific ELISA. G) Transmission electron micrographs of pancreatic islet cells isolated from beta-C3-KO or C3-flox mice. Beta-C3-KO mouse beta-cells contain accumulated amounts of swollen ER or similar vesicular organelles (black arrows), while normal ER is apparent in C3-flox mouse islet cells (white arrows). See also Supplementary Fig. 1. Increased amounts of autophagic material were also observed in beta-C3-KO cells (asterisk). H) *In vitro* insulin secretion from isolated islets from C3-flox or beta-C3-KO mice HFD-fed mice, at 15 or 60 min. I) Average insulin content values per islet from secretion experiment in panel (H) (average values shown for each of 6 mice: from 6 wells, 5 islets per well). J) Serum C3 levels in C3-flox or beta-C3-KO mice at the endpoint of HFD, as measured by ELISA. Statistics in E, one-way ANOVA, in F/H, two-way ANOVA, in I, Student’s T-test, and in B/C, by mixed model statistical analysis (see methods).

At the end of the study, pancreata were removed and sections were stained for glucagon and insulin to assess islet size and structure (Figure 6A). Due to the loss of some samples during preparation and thus lower power, a linear mixed model was used to analyze the results. This analysis showed a positive association between islet size and HFD, where islet size increased by 1.3% per 1% fat added to the diet, 95% CI (lower bound: 0.25%, upper bound: 2.4%). This HFD-induced compensatory increase in islet size is consistent with previously published data [38]. Overall islet size was not affected by genotype (Figure 6B). The beta-C3-KO genotype had a positive association for the overall proportion of each islet staining positive for insulin, where the staining was estimated to increase by 0.063 (9.4% more than the average insulin staining of the control group) in beta-C3-KO mice compared to the C3 flox control mice, 95% CI (lower bound: 0.00935, upper bound: 0.118), independently of diet (Figure 6C), suggesting that C3 plays a role in regulating insulin content or beta-cell numbers.

To investigate potential increases in beta-cell stress induction, we also then stained the same islets with an antibody specific for pro-insulin (Figure 6D). When undergoing ER stress, beta-cell insulin processing becomes less efficient, leading to increased pro-insulin content and secretion, indicative of type 2 diabetes development [39]. Larger areas of islets from beta-cell specific beta-C3-KO mice on HFD stained positive for pro-insulin, compared to flox HFD or beta-C3-KO LFD controls (Figure 6E). While control mice had crescent-patterned pro-insulin staining consistent with the ER and Golgi, beta-C3-KO HFD mice had larger proportions of the cell area stained positive for pro-insulin, consistent with it entering later stages of the secretory pathway. Consistent with this, an ELISA specific for unprocessed pro-insulin also showed increased levels of pro-insulin within the serum of beta-C3-KO mice on HFD, compared to control groups, which became statistically significant by 23 weeks of HFD (Figure 6F).

To further investigate signs of ER stress, islets isolated from beta-C3-KO and C3-flox controls were imaged by transmission electron microscopy. Beta-cells from beta-C3-KO mice contained enlarged intracellular vesicular organelles, (Figure 6G and Supplementary Fig. 1) consistent with appearance of swollen ER that is indicative of ER stress [40] and that can be induced in beta-cells for example by exposure to palmitate [41]. While C3-flox islet cells contained ER of normal appearance, beta-C3-KO cells contained increased amounts of swollen lamellar-like organelles, as well as increased numbers of autophagosomes, identifiable by cytosolic contents surrounded by double membranes (Figure 6G). This observation is consistent with increased ER stress and autophagic content in these cells.

Finally, pancreatic islets were also isolated from C3-flox and beta-C3-KO mice after 6 months of HFD, and insulin secretion tested in response to incubation with either low (2.8 mM or high (16.7 mM) glucose. Insulin secretion from the beta-C3-KO islets was significantly higher than that from control floxed islets (Figure 6H), although we did not find a significant difference in total insulin per islet (Figure 6I). To verify that the differences in observed phenotypes was due to a difference in intrinsic and not circulating C3, C3 was measured in serum samples, confirming that, as at baseline (Figure 1D), there was still no difference in circulating C3 levels between C3-flox and beta-C3-KO mice after 6 months of HFD (Figure 6I).

## 4 Discussion

We and others have described upregulation of C3 expression in pancreatic islets of diabetic humans and rodents, as well as in clonal beta-cells under diabetogenic conditions [20,42,43]. Beta-cell intrinsic expression of C3 is protective against cellular stress and apoptosis induced by free fatty acids, high glucose, streptozotocin, and inflammatory cytokines [20,21,42]. In light of this, we created a beta-cell specific C3–KO mouse (beta-C3-KO) to investigate its functions in an *in vivo* setting. While C3-flox control mice and beta-C3-KO mice had identical circulating C3 levels, C3 was lacking from beta-C3-KO islets. When placed on HFD, female beta-C3-KO mice displayed an enhanced diabetic phenotype, with increased weight, adipose tissue inflammation, and increased fasting blood glucose and insulin. Their islets showed not only increased relative insulin staining, but also increased pro-insulin, which was also seen in serum.

C57Bl/6 mice placed on a HFD exhibit islet hyperplasia, and a functional expansion of beta cell mass to compensate for the increased metabolic pressure and demand for insulin [38]. Although some evidence exists that this islet size increase occurs prior to the development of insulin resistance [44], and does not necessarily correlate with increased insulin secretion upon identical glucose challenge [45], longer-term HFD typically leads to hyperinsulinemia, due to increased insulin resistance [46]. Prolonged HFD and an increased insulin production demand place beta-cells under increased strain. This is thought to lead to increased chronic ER stress induction, contributing to beta-cell failure [47].

The beta-C3-KO mice described in this paper demonstrated a larger proportion of insulin staining per islet, independent of diet, which reflects our previous findings in clonal beta-cells, where C3–KO clones also had increased total insulin content [20], attributed to a downregulation of homeostatic autophagy that is involved in recycling of insulin granules [48]. Autophagy can also relieve ER stress in beta-cells in situations of increased insulin demand [49,50], and therefore it is likely that C3-deficient beta-cells experience increased ER stress, as evidenced by increased intracellular pro-insulin content, as well as increased serum levels of pro-insulin (Figure 6), symptoms of beta-cell stress and diabetes risk [39,47]. However, the beta-C3-KO mice only demonstrated a diabetic-prone phenotype when challenged with a HFD. Similarly, mice with a beta-cell specific deficiency of autophagy component ATG7 only show very slight increases in blood glucose while on a normal diet, until crossed onto hyperphagic ob/ob mice, in which case diabetic symptoms in the beta-cell autophagic deficient hyperphagic mice are more severe than in mice from either parental strain [49]. Therefore, some genetic changes that result in a diabetes-prone phenotype only become apparent on a HFD.

One model for the development of T2D is that in a calorie-rich environment, over-production of insulin leads to increased uptake of glucose and storage of energy, leading to obesity and insulin resistance [[51], [52], [53]]. Pharmacological inhibition of insulin secretion can lead to weight loss in human trials [51] and genetic ablation of one of the insulin genes (e.g *Ins1*) in female mice protects against weight gain on HFD [54,55], without resulting in any apparent phenotype on normal diet. In particular, inducible partial silencing of insulin expression in adult mice on HFD led to reduced adiposity and adipose innate immune gene signatures without significantly affecting blood glucose homeostasis [56]. Additionally, a causative role for increased insulin secretion in obesity, independent of insulin resistance, is suggested by functional [57] and genetic data [58] in humans. It is therefore possible that the previously described deficiency in autophagy in C3–KO beta-cells [20], leading to higher islet insulin content and increased insulin release from islets of beta-C3-KO mice can in the presence of HFD, produce the phenotype described here; faster weight gain, obesity, lowered insulin sensitivity, and subsequently increased beta-cell stress. Consistent with this, we previously found that C3–KO clonal beta cells not only had higher insulin content but also released more insulin in glucose-stimulated secretion assays [20]. In addition, we found that isolated C3–KO mouse islets had higher levels of autophagy markers LC3-II and P62 [20]. This is consistent with our findings that C3–KO clonal beta-cells had deficient autophagosome-lysosome fusion [20]. However, it should also be pointed out that our longitudinal data from cohorts of beta-C3-KO mice did not confirm that significant increase in serum insulin preceded increases in insulin resistance, and further work at earlier timepoints of this model will be needed to verify this. We also did not find significantly different insulin content in islets from these mice at the endpoint of the high-fat diet model (Figure 6I), but these data were not corrected for islet size, and similar investigations should be carried out at earlier timepoints, before the induction of HFD-induced hyperplasia. We did however, find increased proportional insulin secretion from the beta-C3-KO islets (Figure 6H).

Finally, our work also demonstrates a significant phenotype in mice specifically lacking C3 within beta-cells, but which had identical extracellular levels of C3 as control floxed mice. We also demonstrated that beta-cells do not take up C3 from the extracellular environment, which indicates a cell-intrinsic role for C3 within beta-cells *in vivo*, consistent with our previous work using gene-altered clonal beta-cells [20,21]. This likely indicates an intracellular role of C3 in beta-cell homeostasis, as beta-cells are exposed to equal amounts of extracellular C3 in both beta-C3-KO mice and floxed controls. Additionally, this suggests that therapeutic inhibitors targeting serum C3 should not be expected to influence beta-cell function.

In conclusion, our findings highlight the critical role of C3 in maintaining beta-cell homeostasis under metabolic stress. Limitations of the study include the single strain of mouse assessed, and that only female mice were used for the 6-month HFD study. Nevertheless, the beta-C3-KO model shows that C3 expression may protect against beta-cell stress and apoptosis under normal conditions, and that its absence during increased metabolic demand, is linked to elevated insulin content and release, increased weight gain, and subsequent metabolic dysfunction. Further work is required to investigate development of HFD-induced ER stress in beta-C3-KO mouse beta-cells. These insights underscore the complexity of C3’s role in diabetes development and suggest that further investigation into its involvement in beta-cell function is warranted to fully elucidate its impact on in which long-term beta-cell health.

## CRediT authorship contribution statement

**Ben C. King:** Writing – review & editing, Writing – original draft, Investigation, Funding acquisition, Formal analysis, Conceptualization. **Lucie Colineau:** Writing – review & editing, Writing – original draft, Investigation, Formal analysis, Conceptualization. **Julia Slaby:** Investigation. **Olga Kolodziej:** Investigation. **Vaishnavi Dandavate:** Investigation. **Robin Olsson:** Formal analysis. **Malin Fex:** Writing – review & editing, Resources, Formal analysis. **Anna M. Blom:** Writing – review & editing, Supervision, Project administration, Funding acquisition, Conceptualization.

## Declaration of competing interest

The authors declare the following financial interests/personal relationships which may be considered as potential competing interests: Anna M Blom reports financial support was provided by Knut and Alice Wallenberg Foundation. Anna M Blom reports financial support was provided by Swedish Diabetes Association Research Foundation. Ben C King reports financial support was provided by Diabetes Wellness Foundation Network Sweden. Ben C King reports financial support was provided by Bo and Kerstin Hjelt Diabetes Foundation. Ben C King reports financial support was provided by Anna-Greta Crafoord’s Foundation. Ben C King reports financial support was provided by Director Albert Pahlsson Foundation for Research and Charity. Ben C King reports financial support was provided by Magnus Bergvall Foundation. Anna M Blom reports financial support was provided by Swedish Research Council. Anna M Blom reports financial support was provided by Swedish Foundation for Strategic Research. If there are other authors, they declare that they have no known competing financial interests or personal relationships that could have appeared to influence the work reported in this paper.

## Data Availability

Data will be made available on request.
