# Supplementary material for: Beta-cell-specific C3 deficiency exacerbates metabolic dysregulation and insulin resistance in obesity
Source: Mol Metab. 2025 Dec 11;103:102302. doi: 10.1016/j.molmet.2025.102302 (PMC12808594; doi:10.1016/j.molmet.2025.102302)
Supplement: Multimedia component 1 [file mmc1.pdf]

Supplementary figure 1:

Expanded/accumulated intracellular vesicular organelles in beta-C3-KO beta-cells

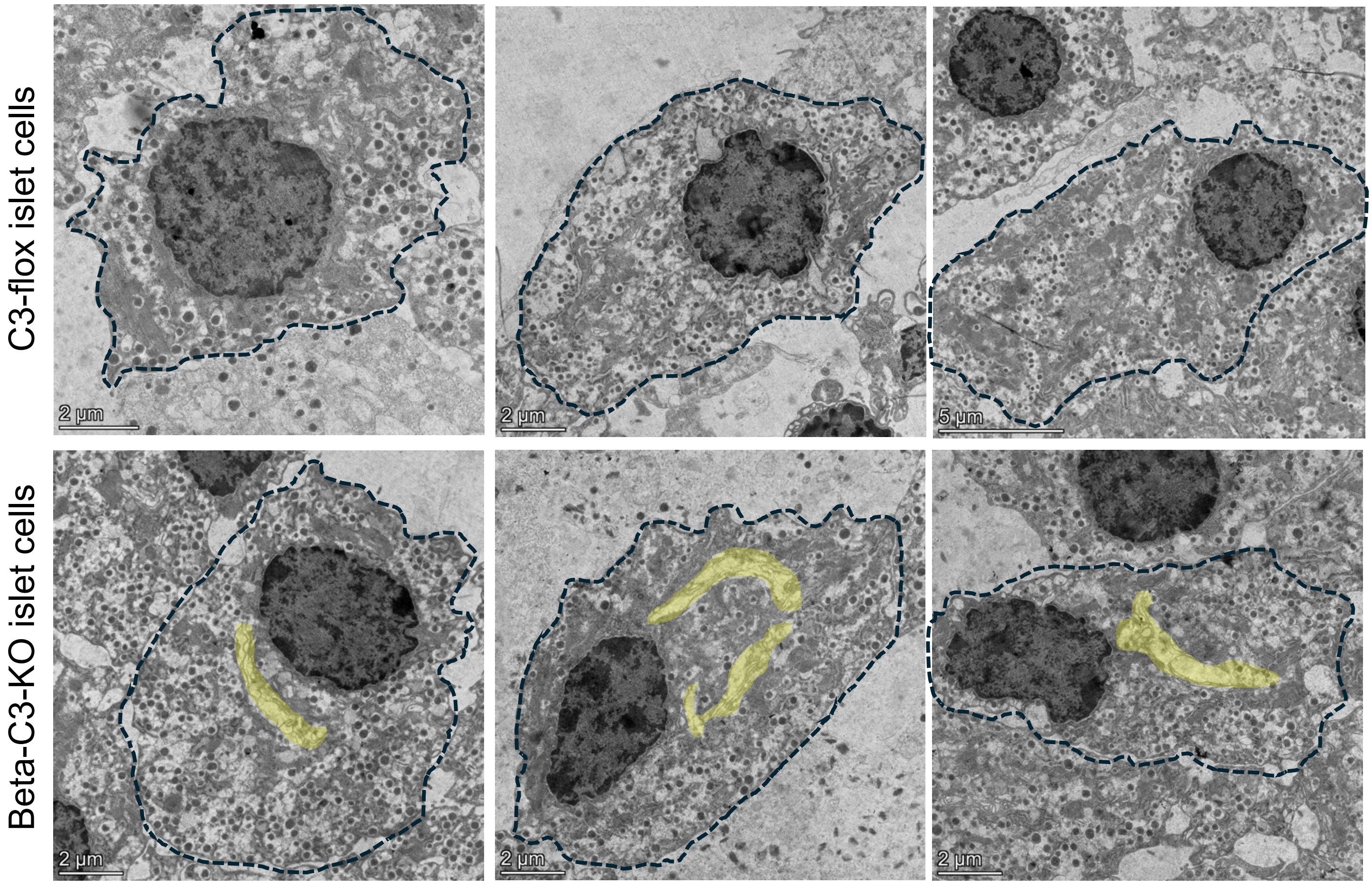

Beta-C3-KO cells without highlights:

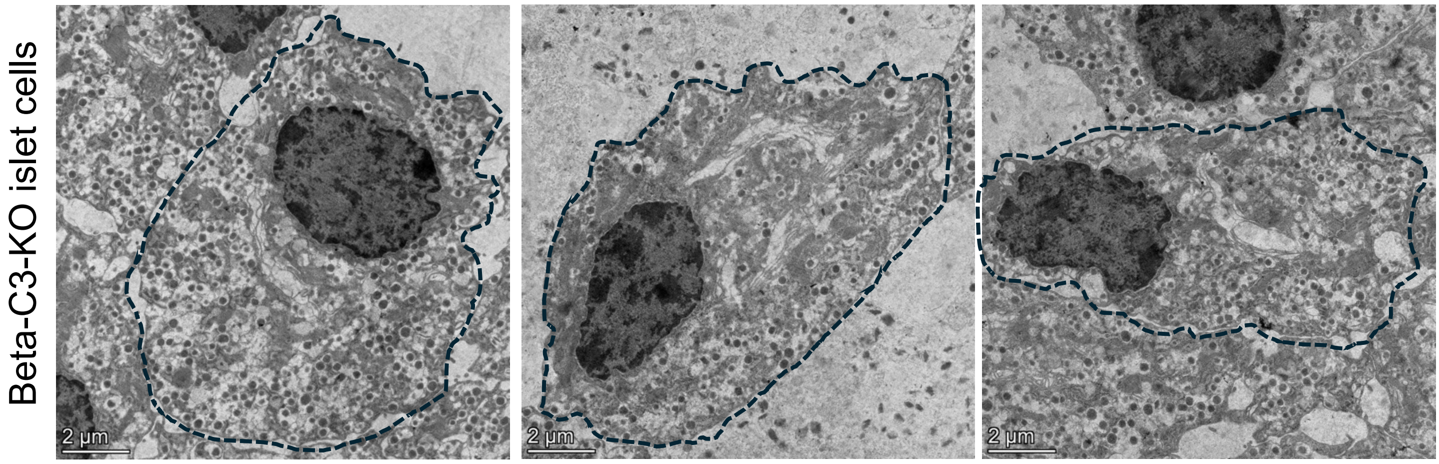

Representative images of beta-cells from pancreatic islets from 3 individual mice per group (C3-flox or beta-C3-KO)

Supplementary figure legend:

Supplementary figure 1: Pancreatic islets were isolated from 6-week old beta-C3-KO or C3-flox mice (n=3 each group), fixed and processed for transmission electron microscopy. Beta-C3-KO islet beta-cells were observed to contain larger vesicular compartments consistent with swollen ER or accumulated autophagosomes, shown highlighted and un-highlighted. Cell membranes are outlined for clarity. Images are from three individual mouse samples for each genotype.
